# Resorcinol-Formaldehyde (RF) as a Novel Plasticizer for Starch-Based Solid Biopolymer Electrolyte

**DOI:** 10.3390/polym12092170

**Published:** 2020-09-22

**Authors:** Vidhya Selvanathan, Mohd Hafidz Ruslan, Mohammod Aminuzzaman, Ghulam Muhammad, N. Amin, Kamaruzzaman Sopian, Md. Akhtaruzzaman

**Affiliations:** 1 Solar Energy Research Institute (SERI), Universiti Kebangsaan Malaysia (UKM), Bangi 43600, Malaysia; hafidzruslan@gmail.com (M.H.R.); ksopian@ukm.edu.my (K.S.); 2 Department of Chemical Science, Faculty of Science, Universiti Tunku Abdul Rahman (UTAR), Perak Campus, Jalan Universiti, Bandar Barat, Kampar 31900, Perak D. R., Malaysia; mohammoda@utar.edu.my; 3 Department of Computer Engineering, College of Computer and Information Sciences, King Saud University, Riyadh 11543, Saudi Arabia; ghulam@ksu.edu.sa; 4 Institute of Sustainable Energy, University Tenaga Nasional (@The National Energy University), Jalan IKRAM-UNITEN, Kajang 43000, Malaysia; nowshad@uniten.edu.my; 5 Centre for Integrated Systems Engineering and Advanced Technologies (Integra), Faculty of Engineering and Built Environment, Universiti Kebangsaan Malaysia (UKM), Bangi 43600, Malaysia; 6 Graduate School of Pure and Applied Sciences, University of Tsukuba, Tsukuba, Ibaraki 305-8573, Japan

**Keywords:** starch, resorcinol-formaldehyde, polymer electrolyte, ionic conductivity, lithium triflate, plasticization

## Abstract

A starch-resorcinol-formaldehyde (RF)-lithium triflate (LiTf) based biodegradable polymer electrolyte membrane was synthesized via the solution casting technique. The formation of RF crosslinks in the starch matrix was found to repress the starch’s crystallinity as indicated by the XRD data. Incorporation of the RF plasticizer improved the conductivity greatly, with the highest room-temperature conductivity recorded being 4.29 × 10^−4^ S cm^−1^ achieved by the starch:LiTf:RF (20 wt.%:20 wt.%:60 wt.%) composition. The enhancement in ionic conductivity was an implication of the increase in the polymeric amorphous region concurrent with the suppression of the starch’s crystallinity. Chemical complexation between the plasticizer, starch, and lithium salt components in the electrolyte was confirmed by FTIR spectra.

## 1. Introduction

Ever since polymer electrolytes were identified as a potential conducting material in the application of highly advanced electrochemical devices, various synthetic polymers have been experimented on by researchers to fit the purpose [1,2]. However, with the current global concern for ecological sustainability, along with the electronic, physical, and thermal credibility of the polymer electrolyte of choice, its biodegradability is another salient point to be taken into consideration [3]. As a plant-derived source would best serve this purpose aptly, this study focuses on the employment of potato starch in developing a highly conductive yet sustainable polymer electrolyte.

Extracted from renewable sources, starch, which belongs to the polysaccharide family, had been acknowledged as a promising alternative to replace the synthetic polymers in various industrial applications. The matrix-forming capacity and complete biodegradability of this low-cost agricultural commodity had motivated its application in the electrochemical field as well [4,5,6].

Despite its chemical, financial, and ecological virtues, starch’s high crystallinity emerges as a major constraint that hinders optimum ionic conductivity in polymer electrolytes synthesized from the biopolymer [7,8,9]. Being the storage unit in plants, starch is naturally found in the form of semicrystalline granules containing amylose and amylopectin in a highly ordered arrangement owing to strong hydrogen bonding between the components [10]. Upon gelatinization, these granules rupture due to water absorption, which, in turn, disrupts the crystalline structure. However, when the gelatinized starch solution is allowed to rest, especially throughout the drying process during film formation, retrogradation occurs as the starch molecules begin to realign themselves [11]. This often leads to the formation of thin, brittle films unfit for polymer electrolyte fabrication.

In order to tackle the crystallinity and retrogradation issues of native starch, the biopolymer is often incorporated with plasticizers to improve the overall flexibility and mechanical properties of the films. Typically, starch films are plasticized with polyols such as sorbitol, glycerol, and ethylene glycol or monosaccharides such as glucose, fructose, galactose, and mannose. The presence of multiple hydroxyl groups in polyols allows hydrogen bond formation with amylose and amylopectin in starch, whereas the structural similarity of monosaccharides enables better compatibility between the polymer and the plasticizer molecule [12]. In recent findings, it has been indicated that hyper-branched polymers can serve as a better plasticizing system for starch as they provide flexibility and concurrently inhibit moisture absorption in the films. Zhang et al. reported the use of hyperbranched poly(citric polyethylene glycol) as novel plasticizers for maize starch-based films [13]. In their work, it was demonstrated, that apart from the hydrogen bond-forming ability, such hyperbranced polymer plasticizers impose a physical entanglement of both starch and plasticizer chains and creates steric hindrance, which more effectively inhibits retrogradation.

This study is a unique attempt to introduce resorcinol-formaldehyde (RF) polymer resin into the system as a means to suppress the crystallinity of starch arising from retrogradation. Resorcinol-formaldehyde has the unique property of forming sol-gel through condensation-polymerization reaction [14]. The formation of an interconnected structure of the RF amongst the starch matrix is expected to mimic a similar effect as the hyperbranced polymers. In addition, the formation of RF-crosslinked clusters throughout the starch matrix will impede the reorganization of amylose and amylopectin, thus suppressing the crystalline nature. Another advantageous property of the RF structure, compared to the typical polyol and monosaccharide-based plasticizers, is the presence of nucleophilic aromatic rings, which will also serve as an active site for ionic complexation and aid in the coherent movement of the ions throughout the polymer matrix. This will boost the ionic conductivity of the polymer electrolyte without the inclusion of high salt content, ensuring the biodegradability of the system is not compromised for its electrical efficiency.

Lithium triflate (LiCF_3_SO_3_) was chosen to be the ionic species in this study. The choice of this salt is justified by its low lattice energy, small polarizable cation, and large, singly charged anion [15]. All these factors promote absolute dissociation of the ionic salt, establishing strong polymer–salt complexes. Besides, the large anionic species may further assist in hampering the polysaccharides’ crystallinity by creating structural disorderliness [16]. Preparation of such lithium ion-doped polymer electrolyte films from natural polymers will also enable prospective applications in lithium-ion batteries [17,18].

Thus, herein, we report the plasticizing effect of the RF solution on the physical and chemical properties, morphology, crystallinity, and ionic conductivity of the starch-based polymer electrolyte system.

## 2. Materials and Methods

### 2.1. Materials

Biopolymer, potato starch, and sodium carbonate were purchased from Omya Hamburg (Germany) and Friendemann Schmidt (Germany). The ionic salt, lithium triflate, and the plasticizer’s component, resorcinol, were acquired from Sigma Aldrich (Germany), whereas glycerol and formaldehyde (37% in aqueous) were supplied by Systerm (Malaysia).

### 2.2. Fabrication of Polymer Electrolyte

#### 2.2.1. Preparation of RF Solution

The resorcinol-formaldehyde reaction in this experiment was catalyzed by Na_2_CO_3_ where the molar ratio of R:Na_2_CO_3_:F was fixed at 50:1:100. Appropriate amounts of resorcinol and sodium carbonate were weighed and dissolved with distilled water. Formaldehyde was added to this solution and stirred for 40 min at room temperature. Upon stirring, the RF solution changed from colorless to yellow, and finally to light brown.

#### 2.2.2. Casting and Film Formation

The starch solution required for the fabrication of the polymer electrolyte was prepared by dissolving 0.25 g of potato starch powder in 10 mL distilled water, and this produced a cloudy solution. The starch was then gelatinized by heating the solution at 80 °C, resulting in a colorless and viscous solution. The solution was then allowed to be cooled down to room temperature before suitable amounts of lithium triflate and RF were added to it according to the composition ratio indicated in Table 1. These solutions were then stirred to allow the formation of a homogeneous solution and complexation between its components. Finally, the homogeneous solution was cast into a clean plastic Petri dish and left to air-dry at ambient temperature to form films. Pure starch film and starch film with 15 wt.% glycerol were also prepared via the method described above as control samples.

### 2.3. Characterization

#### 2.3.1. Fourier-Transform Infrared-Attenuated Total Reflection (FTIR-ATR)

A Perkin-Elmer Spectrum 400 FTIR Spectrometer (MA, USA) was used to obtain the FTIR spectra for the samples via the ATR technique to identify possible chemical interactions between the components in the electrolyte. The samples were subjected to wavenumbers ranging from 4000 to 650 cm^−1^ at ambient temperature and a resolution of 4 cm^−1^.

#### 2.3.2. Electrochemical Impedance Spectroscopy (EIS)

Samples incised to suitable sizes were sandwiched between spring-pressured stainless-steel electrodes with a diameter measuring 2.0 cm. The HIOKI 3532-50 LCR Hi-Tester (Japan) was used to record the impedance data of the films between 50 and 5 MHz. The thickness of each film sample was measured with a digital micrometer screw gauge. From the real and imaginary components of the impedance data, the Nyquist plots for each of the sample were constructed and their corresponding ionic conductivity was calculated by applying the equation:

(1) $$\sigma =\frac{L}{R_{b}A}$$

where *L* is the thickness of the sample measured, *A* is the surface area of contact, and *R_b_* is the bulk resistance of the sample. The value of *R_b_* was evaluated based on the Nyquist plot.

#### 2.3.3. X-ray Diffraction (XRD)

The plasticizing effects of RF additives on the crystallinity of the films were studied using X-ray diffraction characterization with a Panalytical X’Pert³ MRD Diffractometer (Malvern, UK) in which samples were scanned at 2θ angles between 5° and 60°.

## 3. Results

### 3.1. Physical Characteristics

**Figure 1:**
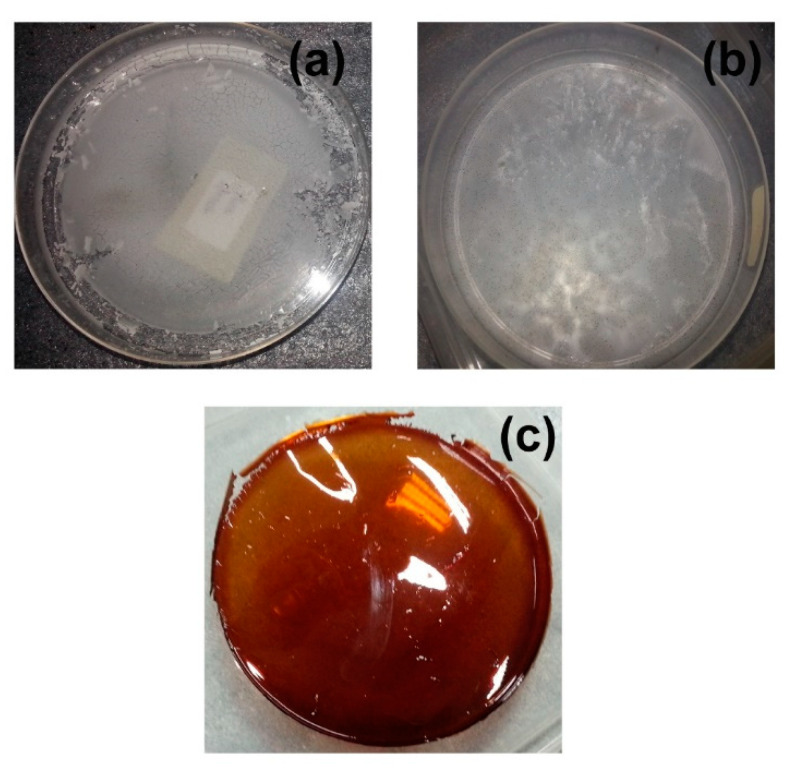
Photographs of (**a**) pure starch film, (**b**) starch film with 15 wt.% glycerol, and (**c**) SRF60-1.

The crystallinity of starch greatly affects its physical appearance, as a highly crystalline structure results in a frail film with poor mechanical properties. As shown in Figure 1a, the casting of gelatinized pure starch film yielded an extremely brittle and fractured film, which was not applicable for EIS analysis. In previous studies on polymer electrolytes based on starch [19], the addition of glycerol was proposed to produce a smoother film, and the inclusion of this plasticizer was indeed found to improve the texture of the film. However, it fails to resolve another major problem occurring with the employment of starch as the polymer host, microorganism growth. 

As a natural food supply, starch films tend to be exposed to fungal growth, especially throughout the drying process. Figure 1b illustrates the growth of fungus on the surface of the glycerol-plasticized starch film. Both these issues of brittleness and fungal attack were efficiently rectified through starch plasticization with RF. This is because both formaldehyde and resorcinol are recognized for their antimicrobial activity against bacteria and fungi and have been applied for endodontic therapy [20,21]. The crosslinking property of RF aids in the formation of a clear, self-standing film (depicted in Figure 1c), which was not susceptible to fungal growth as well. The elastic nature of the film also eased any incision required for characterizations, particularly EIS analysis.

### 3.2. Plasticization Mechanism

**Figure 2:**
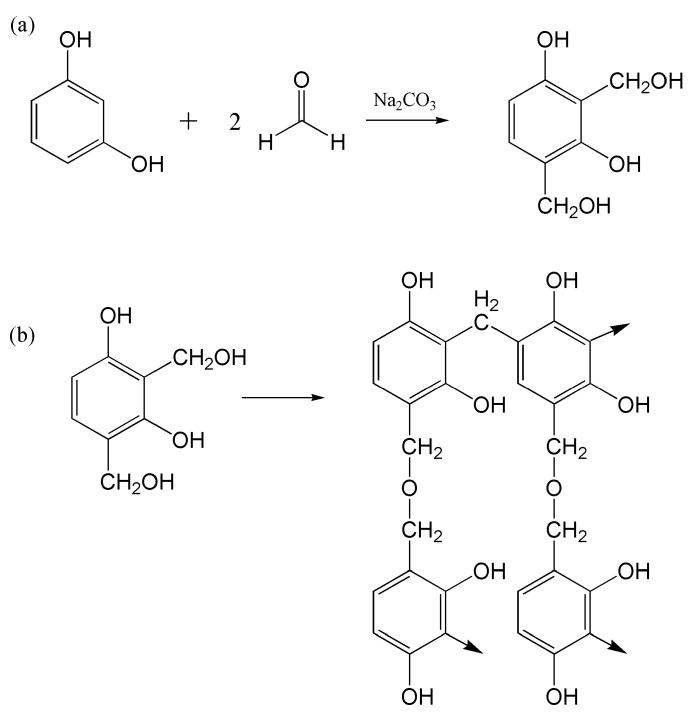
Reaction schematic of RF (**a**) addition reaction and (**b**) polycondensation.

In the presence of a basic medium, in this case, Na_2_CO_3_, resorcinol (R) and formaldehyde (F) of 1:2 molar ratio, goes through an addition reaction to form a hydroxymethyl derivative, as indicated in Figure 2a. The monomers then further undergo polycondensation (Figure 2b) to form extensive crosslinks, and this unique property of RF was manipulated in this study to suppress starch’s crystallinity [14]. As mentioned in Section 2, the resorcinol and formaldehyde were allowed to complete the addition reaction before infusing it into the starch solution. At this stage, the substituted benzene is well-distributed throughout the starch matrix as they are capable of forming hydrogen bonding with starch molecules. Once the solution is cast and allowed to dry, the substituted benzene monomers start crosslinking via condensation, hence interlocking the starch macromolecules. This ensures that the starch structures in the electrolyte membrane do not retrograde, hindering crystallization of the polymer chains.

### 3.3. FTIR-ATR Analysis

**Figure 3:**
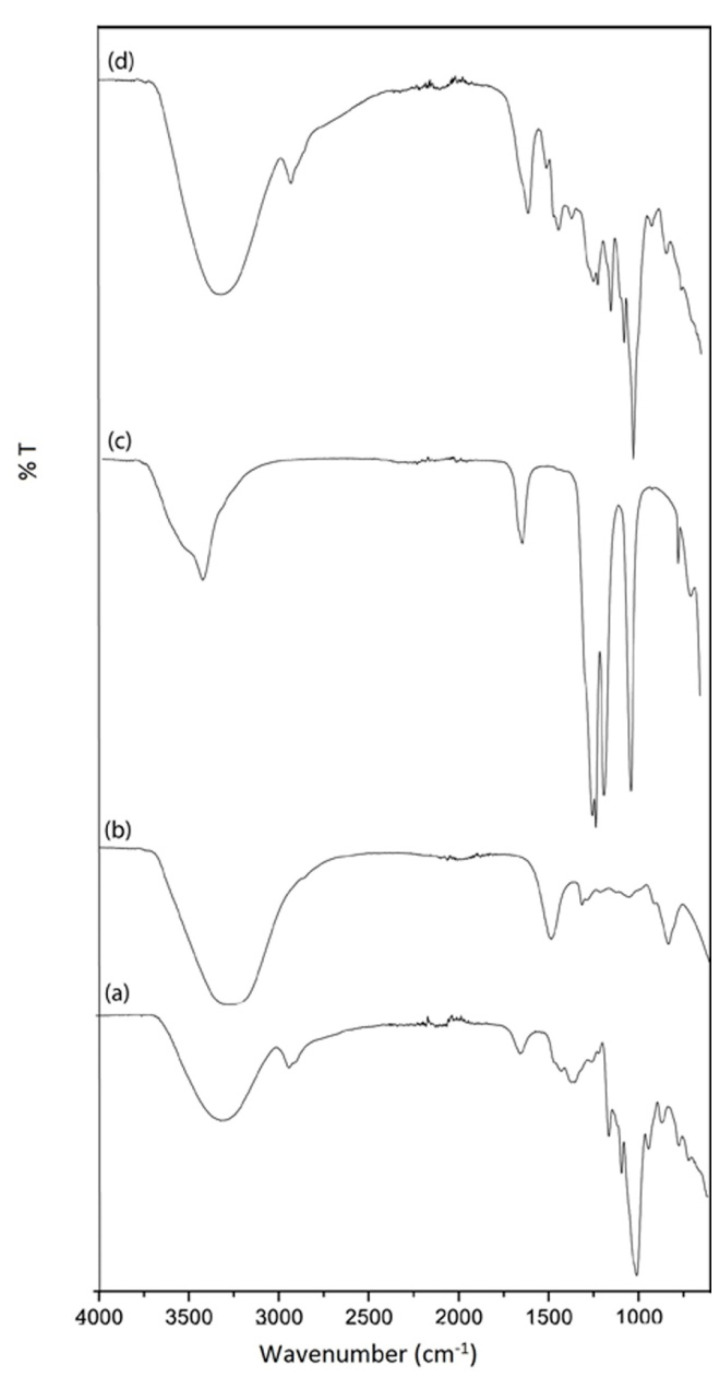
FTIR spectra of the pure constituents: (**a**) starch film, (**b**) RF resin, (**c**) lithium triflate, and (**d**) typical starch/RF/LiTf sample.

The FTIR spectra of pure starch, RF, and lithium triflate are presented in Figure 3. Characteristic absorption peaks of the C–O–C groups at 1150 and 996 cm^−1^ were observed in the pure starch film. The absence of a carbonyl peak in pure RF affirms the complete addition reaction between resorcinol and formaldehyde forming hydroxymethyl derivatives, which are in agreement with previous studies on RF resins.

**Figure 4:**
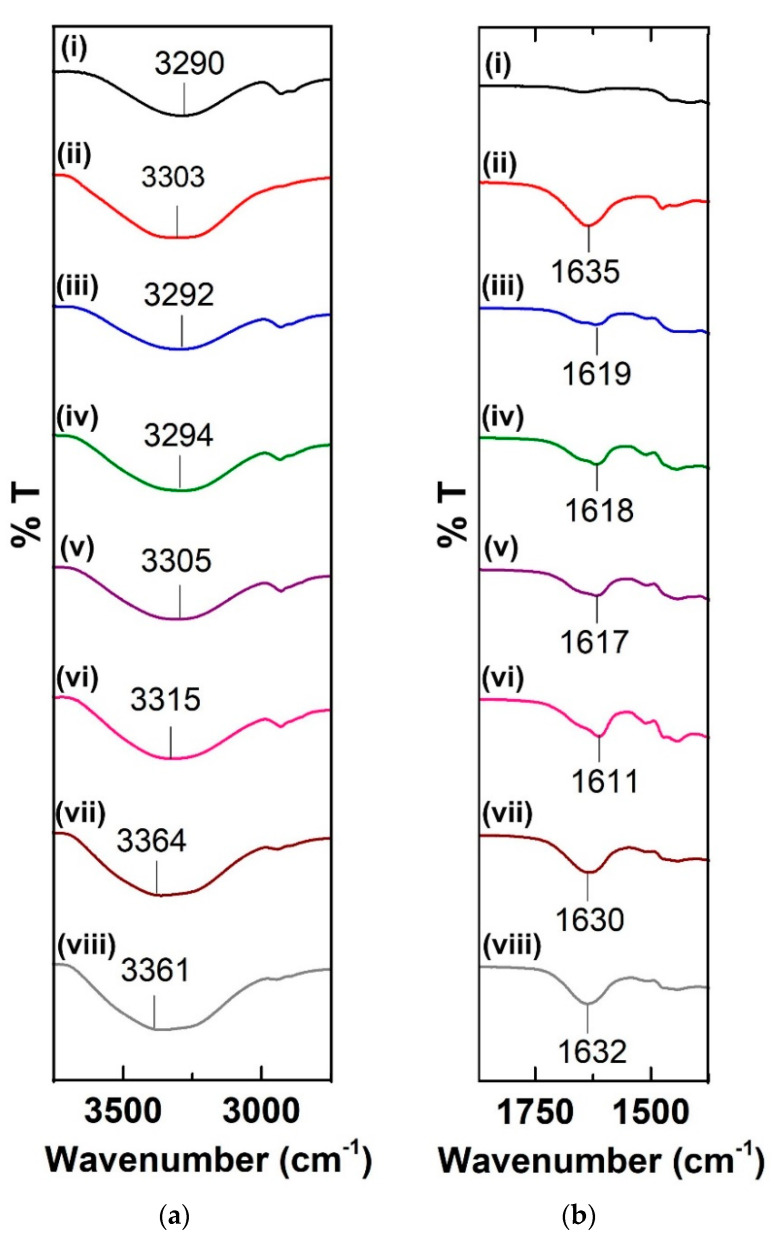
FTIR spectra at (**a**) the hydroxyl region and (**b**) the aromatic region of (i) pure starch film, (ii) RF, (iii) SRF15, (iv) SRF30, (v) SRF45, (vi) SRF60-1, (vii) SRF60-2, and (viii) SRF60-3.

In comparison to the pure constituents, the typical spectrum of the starch/LiTf/RF sample shows drastic transitions at the hydroxyl and aromatic regions. Figure 4a, which shows the FTIR spectra of the samples in the hydroxyl band region, clearly depicts an upward shift pattern, as the amount of RF incorporated is increased. With the addition of 60 wt.% of RF, the hydroxyl band, which initially appeared at 3290 cm^−1^ in the pure starch film, was found to be shifted to 3315 cm^−1^. A higher transition (to 3361 cm^−1^) occurs when the lithium triflate content was increased from 10 to 20 wt.%. The shift to higher wavenumber occurs when more associated hydroxyls become free hydroxyls as a result of restricted hydrogen bonding upon the incorporation of RF into the system [22]. Polymerization-condensation of RF forms a network cluster throughout the polymer matrix, which disrupts extensive hydrogen bonding between the amylose and amylopectin molecules in starch. Steric hindrance created by the RF crosslinking prevents hydrogen bonding within the substituted resorcinol. When the lithium salt content is increased, the possible interaction between the ions and the hydroxyl oxygen further hinders inter- and intramolecular hydrogen bonding.

In comparison to the pure constituents, the typical spectrum of the starch/LiTf/RF sample shows drastic transitions at the hydroxyl and aromatic regions. Figure 4a, which shows the FTIR spectra of the samples in the hydroxyl band region, clearly depicts an upward shift pattern, as the amount of RF incorporated is increased. With the addition of 60 wt.% of RF, the hydroxyl band, which initially appeared at 3290 cm^−1^ in the pure starch film, was found to be shifted to 3315 cm^−1^. A higher transition (to 3361 cm^−1^) occurred when the lithium triflate content was increased from 10 to 20 wt.%. The shift to higher wavenumber occurs when more associated hydroxyls become free hydroxyls as a result of restricted hydrogen bonding upon the incorporation of RF into the system [22]. Polymerization-condensation of RF forms a network cluster throughout the polymer matrix, which disrupts extensive hydrogen bonding between the amylose and amylopectin molecules in starch. Steric hindrance created by the RF crosslinking prevents hydrogen bonding within the substituted resorcinol. When the lithium salt content is increased, the possible interaction between the ions and the hydroxyl oxygen further hinders inter-and intramolecular hydrogen bonding.

As shown in Figure 4b, the complexation between lithium triflate ions and RF was confirmed by the shift at the aromatic C=C region. The interaction between Li^+^ ions and the nucleophilic aromatic ring causes a shift to lower wavenumber in this region, with SRF60-1 exhibiting the largest shift from 1635 (in pure RF) to 1611 cm^−1^. However, increasing the salt content further causes a drop in the value of transition. In SRF60-3, which has a higher lithium salt content, there is only a slight shift, indicating poor complexation between the components, probably due to ion aggregation [23]. Therefore, for this system of electrolyte, the optimum point is at 20 wt.% lithium triflate, after which the performance of the material drops.

### 3.4. XRD Analysis

**Figure 5:**
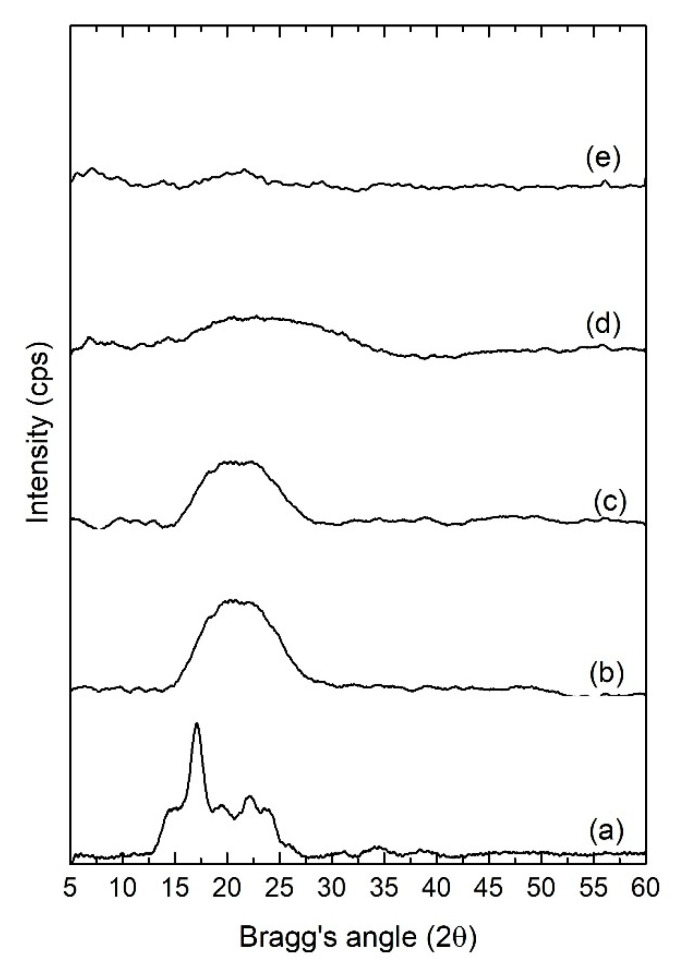
XRD diffractograms of (**a**) pure starch film, (**b**) SRF30, (**c**) SRF45, (**d**) SRF60-1, and (**e**) SRF60-2.

The X-ray diffractograms of pure starch films and selected plasticized films are presented in Figure 5. The pure starch film displayed minor peaks at 2θ values of 17.0°, 19.6°, and 22.1°. These crystalline peaks are indicative of B-type crystallites, which is typical in tuber-originated starches, in this case, potato [24]. As different weight ratios of RF are included in the starch matrix, these peaks were observed to gradually broaden, as demonstrated in Figure 5. Upon addition of 60 wt.% of RF, the minor peaks between 15–25° 2θ regions had disappeared completely, resulting in a broad peak typical of an amorphous sample. Such a trend verifies the ability of RF plasticizer to restrict interchain hydrogen bonding between starch polymers, which, in turn, diminishes the overall crystallinity of the films. In solid polymer electrolyte systems, decreased crystallinity translates into enhanced ionic conductivity as ionic mobility within the electrolyte is facilitated in an amorphous domain with more flexible polymer segments [25]. The increase in lithium triflate concentration from 10 wt.% in SRF60-1 to 20 wt.% in SRF60-2, however, did not produce any significant difference in the diffraction peaks. The absence of any peak corresponding to lithium triflate crystals further validates the complete dissociation of the ions in the polymeric matrix.

### 3.5. Conductivity Studies

The electrical efficiency of the sustainable polymer electrolyte fabricated from starch-RF-lithium triflate was determined via electrochemical impedance data, and the ionic conductivity value corresponding to each sample is presented in Table 2. As the amount of RF added increased, the semicircle region in the Nyquist plots of the samples was found to be depressed, eventually leaving only the inclined line for 60 wt.% of the RF sample. Such a trend of the Nyquist plot had been previously reported [26], and it is an implication of the diminishing capacitive nature with the increase in the plasticizer concentration, where only the resistive component owing to mobile ions remains to exist.

A clear rise in ionic conductivity values was noticed with the increase in RF concentration. Generally, a pure starch film is capable of producing a conductivity value in the magnitude range between 10^−10^ and 10^−9^ S cm^−1^ [19,27]. By including 60 wt.% of RF, this value had been improved up to 10^−5^ S cm^−1^, giving an almost five times higher order of magnitude. Previous studies of starch-based polymers with different lithium salts (LiI and LiClO_4_) reported an ionic conductivity up to 10^−4^ S cm^−1^ only when the salt content was as high as 30–40 wt.% [19,23]. In this starch/LiTf/RF system, a maximum ambient temperature conductivity of 4.29 × 10^−4^ S cm^−1^ can be achieved with only 20 wt.% LiTf. Such ability of RF plasticized starch to generate high ionic conductivity with low salt content consents the material to be more cost-effective and eco-friendly. This was achieved by the incorporation of RF, which, as supported by the XRD and FTIR analyses, suppresses the crystallinity of the starch and creates a new mode for ionic mobility. As depicted in the X-ray diffractograms earlier, a significant decline in crystallinity was observed in SRF60-1, and with the increment in salt content to 20 wt.%, the degree of crystallinity in SRF60-2 further dropped. This facilitated the segmental movements of starch chains within the film and was accompanied by the concurrent increase in the concentration of ionic species present. Ionic conductivity is the product of both mobility and number of ions in the material. Thus, in SRF60-2 film, when both these factors were elevated by the optimized addition of plasticizer and salt, the highest ionic conductivity of 4.29 × 10^−4^ S cm^−1^ was recorded compared to other starch films. The ionic conductivity attained in this study is one of the highest recorded among other plasticized starch film-based polymer electrolytes (Table 3). When the salt concentration was increased from 20 to 30 wt.%, the conductivity declined to a magnitude of 10^−6^ S cm^−1^ owing to the poor mechanical property and formation of salt aggregates [28]. Formation of RF crosslinks throughout the polymeric matrix forces salt ions in high-salt-containing samples to exist as neutral pairs and ion triplets, contributing to the massive drop in conductivity.

**Figure 6:**
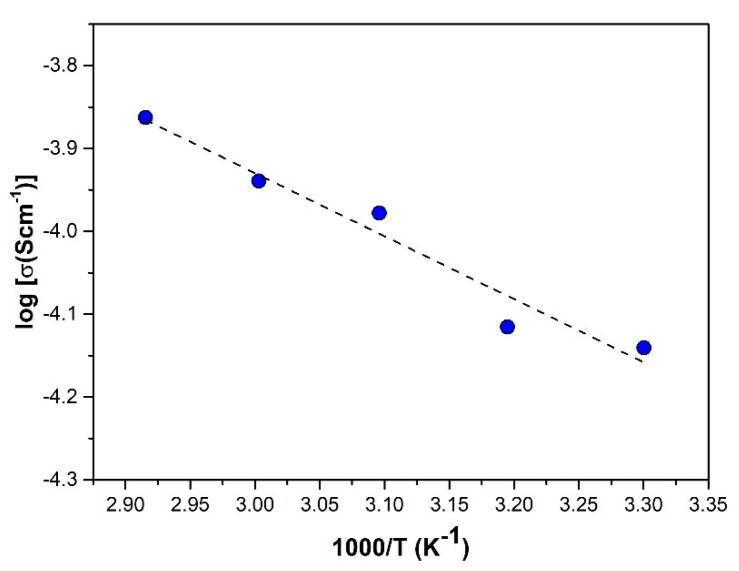
Plot of log conductivity as a function of temperature for SRF60-2.

In order to further comprehend the conducting mechanism of this polymer electrolyte system, the sample with the best mechanical stability and ionic conductivity, SRF60-2, was subjected to temperature-dependent conductivity studies. In the starch-based system, the temperature-dependent conductivity is slightly distinctive because it is governed by two antagonistic factors. Firstly, just as in a conventional polymer electrolyte, an increase in temperature promotes a higher pace of polymeric segmental motion, facilitating ion migration. Hence, conductivity typically increases with temperature. However, as starch possesses a water-absorbing quality and water contributes to charge transport, increasing the temperature will reduce the water content in the system, which, in turn, decreases conductivity. As shown in Figure 6, there was a slight deviation in the increasing conductivity value as the temperature was raised. This may be inferred as the consequence of these two contending factors. Nevertheless, the conductivity–temperature interaction of the SRF60-2 sample did obey the Arrhenius rule, and an activation energy *E*_a_ of 0.15 eV was recorded by the electrolyte.

In preceding studies, a minimum activation energy ranging between 0.4 and 0.5 eV has been monitored in systems consisting of starch-NH_4_NO_3_ [26], starch-LiI [27], and starch-chitosan LiClO_4_ [34]. The value obtained in this study is comparable to another plasticized system, starch-glycerol-LiI [19], which recorded 0.16 eV. Based on the Anderson–Stuart model, the cumulative activation energy of ion conduction constitutes electrostatic binding energy, which is the energy required to cleave the bonding of the ion to its original site and strain energy, which is the energy used to displace the ion from one point to another [35]. Thus, it is proposed that the smaller value recorded in this system can be attributed to the inclusion of RF plasticizers, which accommodates alternative pathways for easy ion movement, hence minimizing strain energy.

### 3.6. Equivalent Circuit Analysis

**Figure 7:**
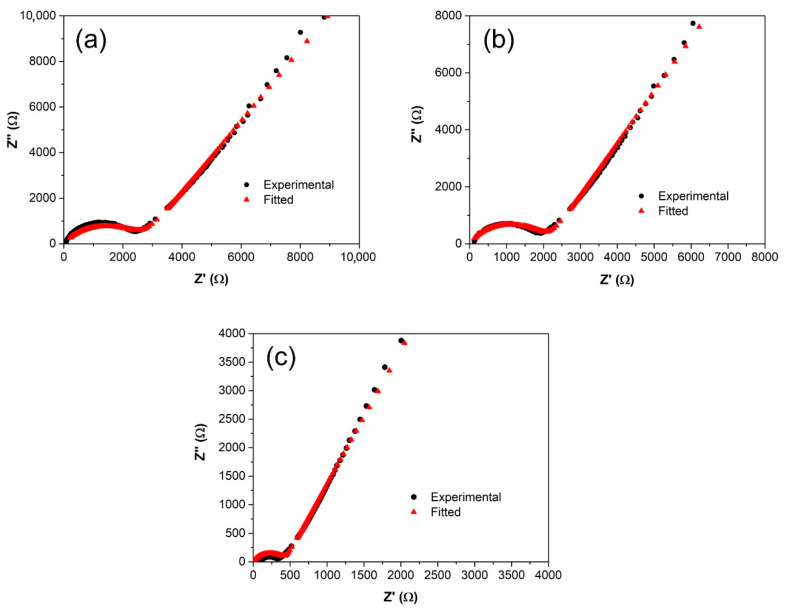
Nyquist plots of (**a**) SRF15, (**b**) SRF30, (**c**) SRF45, and (**d**) the corresponding equivalent circuit.

As shown in Figure 7, the Nyquist plots for SRF15, SRF30, and SRF45 depict a depressed semicircle followed by a sloped spike, which is represented by Rb and constant phase element (CPE) in parallel arrangement connected in series to another CPE element. Equations (1) and (2) highlight the real and imaginary impedance values corresponding to the described equivalent circuit.

(2) $$Z_{r}=\frac{R_{b}+R_{b}^{2}C_{1}\omega^{p_{1}}\cos(\frac{\pi p_{1}}{2})}{1+2R_{b}C_{1}\omega^{p_{1}}\cos(\frac{\pi p_{1}}{2})+R_{b}^{2}C_{1}\omega^{p_{1}}}+\frac{\cos(\frac{\pi p_{2}}{2})}{C_{2}\omega^{p_{2}}}$$

(3) $$Z_{i}=\frac{R_{b}^{2}C_{1}\omega^{p_{1}}\sin(\frac{\pi p_{1}}{2})}{1+2R_{b}C_{1}\omega^{p_{1}}\cos(\frac{\pi p_{1}}{2})+R_{b}^{2}C_{1}\omega^{p_{1}}}+\frac{\sin(\frac{\pi p_{2}}{2})}{C_{2}\omega^{p_{2}}}$$

where *ω* is the angular frequency, *R_b_* is the bulk resistance, *C*_1_ and *C*_2_ denote high- and low-frequency capacitance, respectively, *p*_1_ represents the depression of the semicircle, and *p*_2_ is the parameter related to the tilted angle of the spike. Each of these parameters are summarized in Table 4.

**Figure 8:**
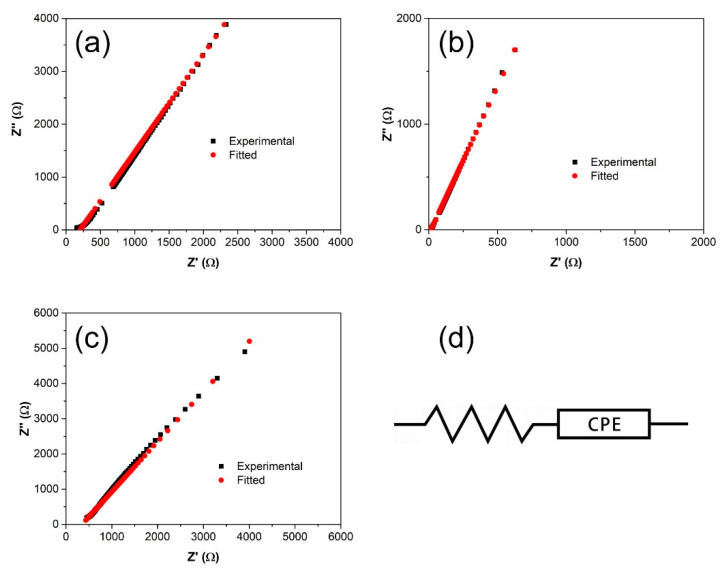
Nyquist plots of (**a**) SRF60-1, (**b**) SRF60-2, (**c**) SRF60-3, and (**d**) the corresponding equivalent circuit.

Upon plasticization up to 60 wt.%, the Nyquist plots of the films only show a tilted spike with complete disappearance of the semicircle (Figure 8). Such a trend is frequently observed in highly conductive polymer electrolytes, in which the equivalent circuit is represented by Rb and CPE connected in series. In this case, the real and imaginary impedance values can be equated to the following equations:

(4) $$Z_{r}=R+\frac{\cos(\frac{\pi p}{2})}{C\omega^{p}}$$

(5) $$Z_{r}=\frac{\sin(\frac{\pi p}{2})}{C\omega^{p}}$$

where *ω* is the angular frequency, *R* and *C* are the resistance and capacitance, respectively, and *p* is the parameter related to the tilted angle of the spike. Table 5 lists these equivalent circuit parameters.

## 4. Conclusions

A series of starch-RF-LiTf-based polymer electrolyte membranes were synthesized by solution casting where starch:LiTf:RF with the composition of 1:1:3 weight ratio recorded the highest room-temperature conductivity of 4.29 × 10^−4^ S cm^−1^. The inclusion of the RF plasticizer was found to repress the starch’s crystallinity as indicated by the XRD data. Therefore, the ionic conductivity was greatly enhanced as an implication of the increase in polymeric amorphous region parallel with the suppression of the starch’s crystallinity. The temperature-dependent study of the sample with the highest conductivity showed Arrhenian behavior and recorded an activation energy of 0.15 eV. Complexation between the polymer host, salt, and plasticizer in the electrolyte was confirmed by FTIR spectra.

## Figures and Tables

**Table 1:** Composition and the designation of starch:LiTf:resorcinol-formaldehyde (RF) polymer electrolyte.

Designation	Composition of RF Plasticizer (wt.%)	Composition of LiTf (wt.%)
SRF15	15	10
SRF30	30	10
SRF45	45	10
SRF60-1	60	10
SRF60-2	60	20
SRF60-3	60	30

**Table 2:** Conductivity values for respective polymer electrolytes.

Sample	Conductivity (S cm^−1^)
SRF15	1.70 × 10^−6^
SRF30	2.61 × 10^−6^
SRF45	2.33 × 10^−5^
SRF60-1	7.23 × 10^−5^
SRF60-2	4.29 × 10^−4^
SRF60-3	9.45 × 10^−6^

**Table 3:** Comparison of recent literature on plasticized starch-based polymer electrolytes.

Type of Starch	Salt Species	Plasticizer	Ionic Conductivity	Reference
Corn	Lithium acetate (LiOAc)	Glycerol	1.03 × 10^−3^	[29]
Corn	Lithium hexafluorophosphate (LiPF_6_)	1-butyl-3-methylimidazolium hexafluorophosphate (BmImPF6)	1.47 × 10^−4^	[30]
Corn	Lithium perchlorate salt (LiClO_4_)	Silica	1.23 × 10^−4^	[31]
Potato	Lithium trifluoromethanesulfonate (LiCF_3_SO_3_)	Graphene oxide (GO)/ 1-butyl-3-methylimidazolium chloride ([Bmim][Cl])	4.80 × 10^−4^	[32]
Corn	Sodium chloride (NaCl)	1-hexyl-3-methylimidazolium iodide (HmIMI)	3.40 × 10^−4^	[33]
Potato	LiCF_3_SO_3_	Resorcinol-formaldehyde	4.29 × 10^−4^	*This work.*

**Table 4:** Equivalent circuit parameters of low-conductivity films.

Sample	*R_b_* (Ω)	*C*_1_ (F)	*p*_1_	*C*_2_ (F)	*p*_2_
SRF15	2620	2.36 × 10^−8^	0.64	1.63 × 10^−6^	0.64
SRF30	1950	4.73 × 10^−8^	0.70	2.17 × 10^−6^	0.68
SRF45	422	6.82 × 10^−8^	0.74	3.40 × 10^−6^	0.72

**Table 5:** Equivalent circuit parameters of high-conductivity films.

Sample	*R_b_* (Ω)	*C* (F)	*p*
SRF60-1	202	2.58 × 10^−^^6^	0.68
SRF60-2	15	6.19 × 10^−^^6^	0.78
SRF60-3	350	4.97 × 10^−^^7^	0.61
